# Umbilical Neutrophil Gelatinase-Associated Lipocalin Level as an Early Predictor of Acute Kidney Injury in Neonates with Hypoplastic Left Heart Syndrome

**DOI:** 10.1155/2015/360209

**Published:** 2015-01-28

**Authors:** Piotr Surmiak, Małgorzata Baumert, Małgorzata Fiala, Zofia Walencka, Andrzej Więcek

**Affiliations:** ^1^Department of Neonatology, Chair of Gynaecology and Obstetrics, School of Medicine in Katowice, Medical University of Silesia, Medykow 14, 40-752 Katowice, Poland; ^2^Department of Nephrology, Endocrinology and Metabolic Diseases, School of Medicine in Katowice, Medical University of Silesia, Francuska 20/24, 40-027 Katowice, Poland

## Abstract

Acute kidney injury (AKI) is a primarily described complication after unbalanced systemic perfusion in neonates with congenital heart defects, including hypoplastic left heart syndrome (HLHS). The aim of the study was to compare the umbilical NGAL concentrations between neonates born with HLHS and healthy infants, as well as to analyze whether the determination of NGAL level could predict AKI in neonates with prenatally diagnosed HLHS. Twenty-one neonates with prenatally diagnosed HLHS were enrolled as study group and 30 healthy neonates served as controls. Perinatal characteristics and postnatal parameters were extracted from the hospital neonatal database. In umbilical cord blood, we determined plasma NGAL concentrations, acid base balance, and lactate and creatinine levels. In neonates with HLHS, complications (respiratory insufficiency, circulatory failure, NEC, IVH, and AKI) were recorded until the day of cardiosurgery. We observed in neonates with HLHS higher umbilical NGAL levels compared to controls. Among 8 neonates with HLHS and diagnosed AKI stage 1, we observed elevated NGAL levels in comparison to those newborns without AKI. Umbilical NGAL could predict, with high sensitivity and specificity, AKI development in study neonates. We suggest that the umbilical blood NGAL concentration may be an early marker to predict AKI in neonates with HLHS.

## 1. Introduction

Hypoplastic left heart syndrome (HLHS) accounts for 1–4% of all congenital heart defects (CHD) [1, 2]. Nevertheless, it is the most common cause of death resulting from cardiac anomalies in the first month of life [2, 3]. Recent approaches focused on understanding and decreasing the frequency of morbidity and complications, including acute kidney injury (AKI) in children with CHD [4]. AKI is an independent predictor of mortality in critically ill neonates and children [5, 6]. The currently available data suggest a trend towards reduced mortality and better renal recovery with earlier initiation of prevention procedures such as monitoring and fluid balance in the subclinical stage of kidney injury [7, 8].

The definition of AKI in a neonate has changed in recent years, and detection is now mostly based on monitoring serum creatinine levels, with or without urine output [9].

Serum creatinine-based definitions fail to delineate the severity, timing, and cause of renal injury. Furthermore, in the first days of neonatal life, creatinine concentrations reflect maternal values (placental transfer) and that serum creatinine level in neonates depends on gestational and postnatal age [9–14].

The quest to find early markers of AKI in neonates is of vital importance [15].

In particular, NGAL is emerging as an excellent biomarker in urine and plasma, for the early prediction of AKI, for monitoring clinical trials in AKI, and for the prognosis of AKI in several common clinical scenarios [16, 17]. The most promising marker of renal injury/damage is neutrophil gelatinase-associated lipocalin (NGAL), secreted by renal tubular epithelium and excreted in the urine during normal renal function [18–20]. NGAL is a protein that is highly upregulated in the proximal tubules after ischemic AKI [21]. Plasma NGAL levels rise markedly after epithelial damage following ischemic/reperfusion kidney injury in neonates after an episode of perinatal asphyxia [22, 23]. NGAL has also been initially introduced in patients with CHD undergoing cardiosurgery [16, 24].

*Aim of the Study*. The aim of the study was to compare the umbilical plasma NGAL concentrations between neonates born with HLHS and healthy infants, as well as to analyze whether the determination of umbilical NGAL level could be used as an early marker of kidney injury in neonates with prenatally diagnosed HLHS.

## 2. Materials and Methods

Our prospective clinical study was performed from January 2013 to January 2014 in the Department of Neonatology, Medical University of Silesia in Katowice, Poland. The study attained an approval from the Human Ethics Committee of the Medical University of Silesia. Before birth, written parental consent was obtained. Patient care and monitoring were performed as part of the hospital's standard protocol, and no specific intervention, other than the collection of umbilical cord blood, was required for this research.

Preterm neonates (below the 37th week of gestation), children with other congenital abnormalities or chromosomal anomalies, newborns of mothers suffering from diabetes mellitus, hypertension, preeclampsia, and children of multiple pregnancies and metabolic disorders were excluded from the study. We also excluded neonates with evidence of congenital infections, as well as those that were born to mothers with clinical chorioamnionitis.

In the preoperative period, we assessed the incidence of respiratory insufficiency, if the neonate required oxygen therapy administration through a nasal cannula, nasal continuous positive airway pressure or mechanical ventilation, circulatory insufficiency when the neonate required inotropic support, necrotizing enterocolitis (NEC), intraventricular haemorrhage (IVH), and acute kidney injury (AKI) [25, 26]. AKI was defined, in accordance with the Acute Kidney Injury Network (AKIN) criteria, as persistently increased serum creatinine (>1.5 mg/dL) for at least 24 hours or rising values > 0.3 mg/dL from the baseline [9, 27].

### 2.1. Study Group Examination

Among 1,271 neonates born in our unit, we enrolled 21 neonates with prenatally diagnosed HLHS, as the study group. In order to assess the comparison of NGAL levels in plasma of umbilical cord blood, 30 healthy, full-term newborns, residing in the unit during the study, were enrolled as the control group.

We evaluated the correlation between the incidence of complications in the early preoperative period and umbilical NGAL levels in neonates with HLHS. Characteristics of the study and control groups are presented in Table 1.

Among 21 infants diagnosed with HLHS, all children from the first day of life presented signs of circulatory failure, 80% had signs of respiratory insufficiency requiring ventilatory support, 38% of children developed AKI stage 1, 9.5% of neonates had NEC, and 14.3% had IVH.

A similar frequency of preoperative complications such as respiratory insufficiency (62% versus 46%; *P* = 0.18) and circulatory failure (100% versus 80%; *P* = 0.22), NEC (12% versus 15%; *P* = 0.61), and IVH (24% versus 23%; *P* = 0.57) was observed in HLHS neonates presenting AKI as compared with HLHS neonates without AKI. Neither of the neonates with HLHS required preoperative renal replacement therapy. There were no differences in mortality (38% versus 46%; *P* = 0.09) and length of stay in our unit (12 versus 14 days; *P* = 0.73) before cardiac surgery, between HLHS neonates with AKI and without AKI (Table 2).

### 2.2. Laboratory Performances

During childbirth, 5 milliliters of blood was collected from the umbilical artery from the placental side, as a standard protocol of our hospital. Blood morphology with total counts of white blood cells (WBC) and neutrophils (manual method) as well as acid-base balance, lactate, creatinine, and NGAL concentrations were performed in all investigated neonates. Umbilical cord blood samples were centrifuged at 2500 revolutions/minute for 10 minutes, and plasma was then stored until assayed at −70°C, in accordance with the recommendations by BioVendor. Plasma NGAL concentrations were determined using the sandwich enzyme immunoassay for the quantitative measurement of human lipocalin-2 (BioVendor—Laboratorní medicína a.s., Brno, Czech Republic). The detection limit of the assay was 0.02 ng/mL.

### 2.3. Statistical Analysis

Continuous variables were compared using Mann-Whitney or Kruskal-Wallis tests, while categorical variables were compared using Chi-square or Fisher's exact tests. The differences and correlations between different analyzed parameters were performed using Kruskal-Wallis test and Spearman's rank test.

Quantitative variables were presented as median and 95% confidence intervals, whereas qualitative variables were shown as percentages. The validity of umbilical NGAL level as a diagnostic marker was assessed using sensitivity and specificity. Statistical analysis was performed using standard procedures available in STATISTICA 10 (Statsoft Polska Inc.) and MedCalc Software Version 12.7.4. Statistical inferences were based on the level of significance *P* < 0.05.

## 3. Results

We observed significantly higher concentrations of NGAL in umbilical cord blood of children with HLHS compared with controls (91.1 ng/mL, 95% CI: 35.9–152.4 ng/mL versus 31.0 ng/mL, 95% CI: 22.9–36.4 ng/mL; *P* < 0.001). No significant difference was found in the creatinine level of neonates from the study and control groups (0.8 mg/dL, 95% CI: 0.7–1.0 mg/dL versus 0.7 mg/dL, 95% CI: 0.6–0.9 mg/dL; *P* = 0.06). However, a higher total count of neutrophils was detected in the umbilical blood (10.1 versus 5.1 × 10^9^/L; *P* = 0.04), without any significant difference in the total WBC count (15.4 versus 15.2 × 10^9^/L; *P* = 0.51) in newborns with HLHS as compared with the control group.

**Figure 1:**
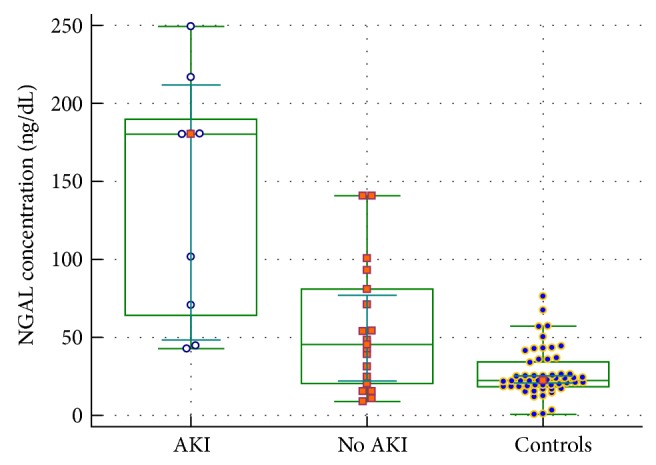
Umbilical NGAL values in three investigated groups of neonates (HLHS-AKI, HLHS-no AKI, and controls). Medians and 95% confidence intervals: maximum and minimum are shown.

**Figure 2:**
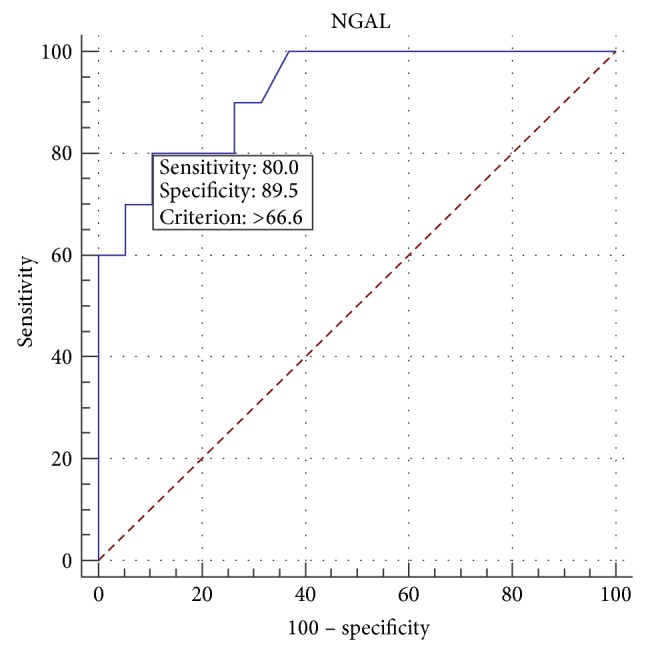
Receiver-operating-characteristic curve is shown for NGAL cord blood concentrations in relation to AKI. Cut-off value for umbilical NGAL 66 ng/dL resulting in a sensitivity of 80% and a specificity of 89.5%^*^ . ^*^ Area under ROC curve, AUC = 0.92 (95% CI: 0.76–0.98; *P* < 0.0001); likelihood ratios, +LR = 7.6 (95% CI: 2.0–29.2); −LR = 0.22 (95% CI: 0.06–0.8).

Furthermore, umbilical NGAL concentrations in neonates with HLHS and diagnosed AKI were significantly higher in comparison to other newborns with HLHS without AKI (171 ng/dL, 95% CI: 66.1–187 ng/dL versus 29 ng/dL, 95% CI: 23–42 ng/dL; *P* < 0.001)—Figure 1. ROC curve analysis demonstrated a critical level of NGAL in the umbilical cord blood > 66.6 ng/mL, and this allows with 80% sensitivity (95% CI: 28–99%) and 89.5% specificity (95% CI: 72–99%) predicting AKI in newborns with HLHS (Figure 2). We also found a significant correlation between umbilical plasma NGAL level and severity of acidosis (lactate (*r* = 0.29; *P* = 0.005) and HCO_3_ concentrations (*r* = −0.25; *P* = 0.02)) in newborns with HLHS. However, there was no correlation between umbilical plasma NGAL and umbilical creatinine concentrations in neonates with HLHS (*r* = −0.11; *P* = 0.23). We also found a correlation between NGAL levels and total neutrophil count in neonates with HLHS (*r* = 0.32; *P* = 0.04) (Table 3). We did not observe any difference in preoperative oxygen saturation between neonates with HLHS and AKI and those without AKI (89%, 95% CI: 79–92% versus 91%, 95% CI: 82–95%; *P* = 0.51). There were no correlations between NGAL level in umbilical cord blood and neonatal mortality (*r* = 0.24; *P* = 0.54) and preoperative complications (NEC (*r* = 0.14; *P* = 0.67), respiratory insufficiency (*r* = 0.53; *P* = 0.36), circulatory insufficiency (*r* = 0.74; *P* = 0.07), or IVH (*r* = 0.67; *P* = 0.37)) in neonates with HLHS.

## 4. Discussion

To the best of our knowledge, NGAL has never been investigated in umbilical cord blood in neonates with prenatally diagnosed HLHS complicated by AKI.

The present study focused on determining whether plasma NGAL concentrations in neonates born with HLHS, measured in umbilical arterial blood, could play an important role in AKI prediction. Literature regarding this topic reveals that acute or chronic dysfunction of the heart or kidneys results from a failure of one of the organs, which then may lead to damage of another organ. This dysfunction is known as cardiorenal syndrome (CRS) [28, 29]. In our opinion, in the case of some children with HLHS, CRS may complicate the course of the disease.

This can be confirmed by elevated levels of umbilical NGAL level in neonates with HLHS compared to the control group and significantly higher levels of plasma NGAL in neonates who developed AKI in the first day of life, despite normal serum creatinine levels. ROC analysis showed high sensitivity and specificity for early diagnosis of kidney injury, by determining the umbilical plasma NGAL concentration in newborns with HLHS.

We believed that AKI in neonates with HLHS could complicate the preoperative period; however, it occurred that complications such as respiratory failure and circulatory insufficiency, NEC and IVH, length of stay in hospital, and mortality were similar in HLHS newborns with and without AKI. Our results opposed those of Theilen and Shekerdemian [30]. These authors suggested that complications such as mesenteric ischemia leading to severe NEC, and hypoxic-ischemic kidney injury or haemorrhagic cerebral damage, can ultimately preclude cardiosurgical intervention.

Absence of a viable left ventricle leads to dilatation and hypertrophy of the right ventricle. The right ventricle experiences an increase in volume load, as blood is diverted away from the underdeveloped left side. Both the systemic and pulmonary vasculatures are perfused via the right ventricle and pulmonary artery, with patency of the ductus arteriosus assuring blood supply to the systemic circulation [31]. The decrement in cardiac output and disturbance in systemic circulation may explain the limitations in somatic growth seen in these fetuses and impaired neurodevelopmental outcome [32–34].

In our opinion, the disturbance in systemic circulation occurring in foetuses with HLHS can also lead to kidney injury. Kidneys normally receive up to 5% of the cardiac output during the fetal period and seem to be very sensitive to hypoperfusion and ischemic insults [35, 36]. Decreased renal blood flow may also be attributable to high renal vascular resistance from an imbalance between renal vasoconstriction and vasodilation, and this may lead to kidney injury, and metabolic acidosis additionally accelerates this process [22, 23, 37, 38]. We have shown that NGAL concentration correlates with the severity of metabolic acidosis (concentration of lactate, HCO_3_, and base deficit) in neonates with HLHS.

Furthermore, we observed that NGAL level correlated with a higher total number of neutrophils in the umbilical blood of neonates with HLHS and AKI, because the increased neutrophil count had been the result of perinatal asphyxia. Morkos et al. showed similar results [39–41].

As a consequence of the relatively small sample size, the statistical power of this comparison between AKI and no-AKI patients is limited. In addition, an absence of consensus over the definition of AKI in neonates complicates comparability between studies.

In conclusion, an increase, especially in cord blood NGAL levels, in neonates with HLHS may be indicative of tubular injury, as a result of CRS, without changes in serum creatinine. This so-called subclinical kidney injury is very difficult to detect. Introduction of appropriate nephroprotective treatment at this stage can prevent the newborn from developing renal insufficiency in the future. It is known that approximately 50% of pediatric patients, who presented AKI in their medical history, showed signs of chronic renal insufficiency [12].

Furthermore, to the best of our knowledge, our study is novel, demonstrating the association between the amount of congenital heart defects with cardiorenal signaling and biomarker of kidney injury. Therefore, confirmation by future prospective multicentre cohorts is needed to assess NGAL concentration, at which one would expect the occurrence of AKI in the first days of life, in order to implement the appropriate treatment as soon as possible.

## Figures and Tables

**Table 1:** Baseline characteristics according to study and control groups (Kruskal-Wallis test, Chi-square test)^*^ .

Study group	Neonates HLHS-AKI (*n* = 8)	Neonates HLHS no-AKI (*n* = 13)	Controls (*n* = 30)
Gestational age (weeks)	37 [37,39]	38 [37,39]	39 [37,40]
Gender: female (%)	3 (35%)	5 (40%)	12 (40%)
Apgar score in 5th min (pts)	8 [7,9]	7 [7,8]	9 [8,9]
Delivery			
Vaginal delivery (%)	0	4 (30%)	10 (35%)
Cesarean section (%)	8 (100%)	9 (70%)	20 (65%)
Blood gas analysis			
pH			
>7.2	5 (62%)	7 (54%)	14 (47%)
<7.2	3 (48%)	6 (46%)	16 (53%)
HCO_3_ (mmol/L)	19.8 [16.1,21.8]	21.4 [20.5,23.1]	23.6 [22.4,24.3]
BE (mmol/L)	−6.8 [−13.4, −3.4]	−5.9 [−7.4, −4.6]	−2.4 [−3.6, −1.6]
Lactate (ng/mL)	6.3 [3.2,9.2]	3.5 [2.5,4.0]	1.9 [1.7,2.2]
Creatinine (mg/dL)	0.9 [0.8,1.1]	0.8 [0.7,0.8]	0.7 [0.6,0.9]
White blood cell count [×10^9^/L]	15.4 [14.0,22.2]	14.8 [9.8,23.2]	15.2 [5.6,20.5]
Total neutrophil count [×10^9^/L]	10.1 [5.1,14.1]	8.9 [5.9,13.4]	5.1 [2.6,10.5]

^*^Results shown as medians and [95% confidence intervals] or percentages.

**Table 2:** Differences between mortality, length of stay, and frequency of complications occurring in the group of neonates with HLHS and AKI in comparison with the group of children with HLHS no-AKI (Mann-Whitney *U* test, Chi-square test)^*^ .

Study group	Neonates HLHS-AKI (*n* = 8)	Neonates HLHS no-AKI (*n* = 13)	*P* value
SaO_2_ before surgery (%)	89 [88–93]	91 [88–95]	0.73
Length of stay in NICU	12 [11–16]	14 [12–18]	0.32
Mortality before procedure (%)	3 (38%)	6 (46%)	0.09
Respiratory failure (%)	5 (62%)	6 (46%)	0.18
Circulatory insufficiency (%)	8 (100%)	10 (80%)	0.22
Necrotising enterocolitis (%)	1 (12%)	1 (8%)	0.57
IVH II (%)	1 (12%)	1 (8%)	0.57
IVH III-IV (%)	1 (12%)	2 (15%)	0.61

^*^Results shown as medians and [95% confidence intervals] or percentages.

**Table 3:** Correlations between selected parameters measured in umbilical cord blood in investigated newborns with HLHS and umbilical NGAL levels (Spearman's rank correlation).

	Creatinine [mg/dL]	Lactate (ng/mL)	HCO_3_ (mmol/L)	Base deficit (mmol/L)	Total neutrophil count (×10^9^/L)
NGAL level [ng/mL]					
Correlation coefficient	0.17	0.29	−0.25	−0.42	0.32
Significance level *P*	0.2	0.005	0.02	<0.001	0.04

## References

[1] Lastinger L., Zaidi A. N. (2013). The adult with a fontan: a panacea without a cure? Review of long-term complications. *Circulation Journal*.

[2] Morris C. D., Outcalt J., Menashe V. D. (1990). Hypoplastic left heart syndrome: natural history in a geographically defined population. *Pediatrics*.

[3] Claxon-McKinney B. (2001). Hypoplastic left heart syndrome. *Pediatric Nursing*.

[4] Pedersen K. (2012). Acute kidney injury in children undergoing surgery for congenital heart disease. *European Journal of Pediatric Surgery*.

[5] Askenazi D. J., Griffin R., McGwin G., Carlo W., Ambalavanan N. (2009). Acute kidney injury is independently associated with mortality in very low birthweight infants: a matched case: control analysis. *Pediatric Nephrology*.

[6] Naik S., Sharma J., Yengkom R., Kalrao V., Mulay A. (2014). Acute kidney injury in critically ill children: risk factors and outcomes. *Indian Journal of Critical Care Medicine*.

[7] Askenazi D. J., Koralkar R., Hundley H. E., Montesanti A., Patil N., Ambalavanan N. (2013). Fluid overload and mortality are associated with acute kidney injury in sick near-term/term neonate. *Pediatric Nephrology*.

[8] Seabra V. F., Balk E. M., Liangos O., Sosa M. A., Cendoroglo M., Jaber B. L. (2008). Timing of renal replacement therapy initiation in acute renal failure: a meta-analysis. *American Journal of Kidney Diseases*.

[9] Jetton J. G., Askenazi D. J. (2012). Update on acute kidney injury in the neonate. *Current Opinion in Pediatrics*.

[10] Koralkar R., Ambalavanan N., Levitan E. B., McGwin G., Goldstein S., Askenazi D. (2011). Acute kidney injury reduces survival in very low birth weight infants. *Pediatric Research*.

[11] Askenazi D. J., Koralkar R., Levitan E. B. (2011). Baseline values of candidate urine acute kidney injury biomarkers vary by gestational age in premature infants. *Pediatric Research*.

[12] Huynh T. K., Bateman D. A., Parravicini E. (2009). Reference values of urinary neutrophil gelatinase—associated lipocalin in very low birth weight infants. *Pediatric Research*.

[13] Bagshaw S. M., Gibney R. T. N. (2008). Conventional markers of kidney function. *Critical Care Medicine*.

[14] Harrison A. M., Davis S., Eggleston S., Cunningham R., Mee R. B. B., Bokesch P. M. (2003). Serum creatinine and estimated creatinine clearance do not predict perioperatively measured creatinine clearance in neonates undergoing congenital heart surgery. *Pediatric Critical Care Medicine*.

[15] Argyri I., Xanthos T., Varsami M. (2013). The role of novel biomarkers in early diagnosis and prognosis of acute kidney injury in newborns. *American Journal of Perinatology*.

[16] Seitz S., Rauh M., Gloeckler M., Cesnjevar R., Dittrich S., Koch A. M. E. (2013). Cystatin C and neutrophil gelatinase-associated lipocalin: biomarkers for acute kidney injury after congenital heart surgery. *Swiss Medical Weekly*.

[17] Askenazi D. J., Feig D. I., Graham N. M., Hui-Stickle S., Goldstein S. L. (2006). 3–5 year longitudinal follow-up of pediatric patients after acute renal failure. *Kidney International*.

[18] Bojan M., Vicca S., Lopez-Lopez V. (2014). Predictive performance of urine neutrophil gelatinase-associated lipocalin for dialysis requirement and death following cardiac surgery in neonates and infants. *Clinical Journal of the American Society of Nephrology*.

[19] Waikar S. S., Bonventre J. V. (2008). Biomarkers for the diagnosis of acute kidney injury. *Nephron Clinical Practice*.

[20] Parikh C. R., Devarajan P., Zappitelli M. (2011). Postoperative biomarkers predict acute kidney injury and poor outcomes after pediatric cardiac surgery. *Journal of the American Society of Nephrology*.

[21] Supavekin S., Zhang W., Kucherlapati R., Kaskel F. J., Moore L. C., Devarajan P. (2003). Differential gene expression following early renal ischemia/reperfusion. *Kidney International*.

[22] Surmiak P., Baumert M., Fiala M. (2014). Umbilical cord blood NGAL concentration as an early marker of perinatal asphyxia in neonates. *Ginekologia Polska*.

[23] Gupta B. D., Sharma P., Bagla J., Parakh M., Soni J. P. (2005). Renal failure in asphyxiated neonates. *Indian Pediatrics*.

[24] Thakar C. V., Worley S., Arrigain S., Yared J.-P., Paganini E. P. (2007). Improved survival in acute kidney injury after cardiac surgery. *American Journal of Kidney Diseases*.

[25] Goldstein B., Zimmerman J. J. (1998). New horizons, the science and practice of acute medicine: critical care of pediatric shock. *Critical Care Medicine*.

[26] Tobias J. D. (2005). Cardiovascular physiology, shock, inotropic agents, and invasive hemodynamic monitoring. *Pediatrics in Review*.

[27] Mehta R. L., Kellum J. A., Shah S. V. (2007). Acute kidney injury network: report of an initiative to improve outcomes in acute kidney injury. *Critical Care (London, England)*.

[28] Ronco C., di Lullo L. (2014). Cardiorenal syndrome. *Heart Failure Clinics*.

[29] Ronco C., Haapio M., House A. A., Anavekar N., Bellomo R. (2008). Cardiorenal syndrome. *Journal of the American College of Cardiology*.

[30] Theilen U., Shekerdemian L. (2005). The intensive care of infants with hypoplastic left heart syndrome. *Archives of Disease in Childhood: Fetal and Neonatal Edition*.

[31] Bohlmeyer T. J., Helmke S., Ge S. (2003). Hypoplastic left heart syndrome myocytes are differentiated but possess a unique phenotype. *Cardiovascular Pathology*.

[32] Mahtab E. A. F., Gittenberger-De Groot A. C., Vicente-Steijn R. (2012). Disturbed myocardial connexin 43 and N-cadherin expressions in hypoplastic left heart syndrome and borderline left ventricle. *The Journal of Thoracic and Cardiovascular Surgery*.

[33] Mahle W. T., Clancy R. R., Moss E. M., Gerdes M., Jobes D. R., Wernovsky G. (2000). Neurodevelopmental outcome and lifestyle assessment in school-aged and adolescent children with hypoplastic left heart syndrome. *Pediatrics*.

[34] Massaro A. N., El-Dib M., Glass P., Aly H. (2008). Factors associated with adverse neurodevelopmental outcomes in infants with congenital heart disease. *Brain and Development*.

[35] Rudolph A. M., Heymann M. A. (1970). Circulatory changes during growth in the fetal lamb. *Circulation Research*.

[36] Xia S., Lv J., Gao Q. (2014). Prenatal exposure to hypoxia induced beclin 1 signaling-mediated renal autophagy and altered renal development in rat fetuses. *Reproductive Sciences*.

[37] Dent C. L., Ma Q., Dastrala S. (2007). Plasma neutrophil gelatinase-associated lipocalin predicts acute kidney injury, morbidity and mortality after pediatric cardiac surgery: a prospective uncontrolled cohort study. *Critical Care*.

[38] Donofrio M. T., Bremer Y. A., Schieken R. M. (2003). Autoregulation of cerebral blood flow in fetuses with congenital heart disease: the brain sparing effect. *Pediatric Cardiology*.

[39] Morkos A. A., Hopper A. O., Deming D. D. (2007). Elevated total peripheral leukocyte count may identify risk for neurological disability in asphyxiated term neonates. *Journal of Perinatology*.

[40] Basile D. P. (2004). Rarefaction of peritubular capillaries following ischemic acute renal failure: a potential factor predisposing to progressive nephropathy. *Current Opinion in Nephrology and Hypertension*.

[41] Kelly K. J., Williams W. W., Colvin R. B. (1996). Intercellular adhesion molecule-1-deficient mice are protected against ischemic renal injury. *The Journal of Clinical Investigation*.

